# The Hfq regulon of *Neisseria meningitidis*

**DOI:** 10.1002/2211-5463.12218

**Published:** 2017-04-25

**Authors:** Robert A. G. Huis in ‘t Veld, Gertjan Kramer, Arie van der Ende, Dave Speijer, Yvonne Pannekoek

**Affiliations:** ^1^ Department of Medical MicrobiologyCenter of Infection and Immunity Amsterdam (CINIMA)Academic Medical CenterAmsterdamThe Netherlands; ^2^ Clinical Proteomics FacilityDepartment of Medical BiochemistryAcademic Medical CenterAmsterdamThe Netherlands; ^3^ Reference Laboratory for Bacterial MeningitisDepartment of Medical MicrobiologyAcademic Medical CenterAmsterdamThe Netherlands; ^4^ Present address: Genome Biology UnitEMBL HeidelbergHeidelbergGermany

**Keywords:** Hfq, mass spectrometry, *Neisseria meningitidis*, proteomics, ribo‐regulation, sRNA

## Abstract

The conserved RNA‐binding protein, Hfq, has multiple regulatory roles within the prokaryotic cell, including promoting stable duplex formation between small RNAs and mRNAs, and thus *hfq* deletion mutants have pleiotropic phenotypes. Previous proteome and transcriptome studies of *Neisseria meningitidis* have generated limited insight into differential gene expression due to Hfq loss. In this study, reversed‐phase liquid chromatography combined with data‐independent alternate scanning mass spectrometry (LC‐MS^E^) was utilized for rapid high‐resolution quantitative proteomic analysis to further elucidate the differentially expressed proteome of a meningococcal *hfq* deletion mutant. Whole‐cell lysates of *N. meningitidis* serogroup B H44/76 wild‐type (wt) and H44/76Δ*hfq* (Δ*hfq*) grown in liquid growth medium were subjected to tryptic digestion. The resulting peptide mixtures were separated by liquid chromatography (LC) prior to analysis by mass spectrometry (MS^E^). Differential expression was analyzed by Student's *t*‐test with control for false discovery rate (FDR). Reliable quantitation of relative expression comparing wt and Δ*hfq* was achieved with 506 proteins (20%). Upon FDR control at *q *≤* *0.05, 48 up‐ and 59 downregulated proteins were identified. From these, 81 were identified as novel Hfq‐regulated candidates, while 15 proteins were previously found by SDS/PAGE/MS and 24 with microarray analyses. Thus, using LC‐MS^E^ we have expanded the repertoire of Hfq‐regulated proteins. In conjunction with previous studies, a comprehensive network of Hfq‐regulated proteins was constructed and differentially expressed proteins were found to be involved in a large variety of cellular processes. The results and comparisons with other gram‐negative model systems, suggest still unidentified sRNA analogs in *N. meningitidis*.

**Abbreviations**
FDR: false discovery rate
Fur: ferric uptake regulator
LC: liquid chromatography
LPS: lipopolysaccharides
MS: mass spectrometry
OMP: outer membrane protein
sRNA: small RNA
TCA: tricarboxylic acid
WT: wild‐type
Zur: zinc uptake regulator

The human pathogen *Neisseria meningitidis* is significant in causing major clinical syndromes such as fulminant septicemia and meningitis. The virulence of *N. meningitidis* results in a high morbidity and mortality rate worldwide regardless of widespread vaccination and available treatment 1, 2.

Ribo‐regulation in prokaryotes uses small antisense RNAs (sRNAs) to regulate the expression of gene systems by RNA–RNA interaction. In recent reviews, the diversity of sRNAs influencing target function expression or mRNA stability, which achieves both target activation and repression has been discussed 3, 4, 5. The widely conserved chaperone protein Hfq is pivotal for ribo‐regulation, regulating metabolic pathways and virulence gene expression 6, 7, 8, 9, 10.

Previously, the role of Hfq as a potential virulence factor in *N. meningitidis* has been assessed by transcriptomic approaches such as (tiling) microarrays 11, 12 or proteomic approaches such as SDS/PAGE protein separation followed by mass spectrometry (MS) 13, 14. The transcriptomic approach has led to the discovery and identification of two Hfq dependent sRNAs as being involved in iron metabolism 15 and survival in oxygen‐limited environments 12. SDS/PAGE protein separation, however, has its limitations in being laborious, having restricted physical resolution, and difficulty in detecting hydrophobic integral membrane proteins and low‐copy number proteins 16, 17, 18, 19. Therefore, only a limited amount of proteins can be differentiated and subsequently identified using MS analysis.

Reversed‐phase liquid chromatography (LC) prior to analysis by data‐independent alternate scanning mass spectrometry (MS^E^) allows for the absolute quantitation of hundreds of proteins in a complex mixture. LC‐MS^E^ can be optimized for a standardized workflow that has a stable performance and efficiency throughout the experiment, facilitating high reproducibility 20, 21, 22. We applied LC‐MS^E^ analysis of differential protein expression in wild‐type vs. Hfq‐deficient meningococcal cells. The differential protein expression may be the result of (a) a direct interaction between Hfq, sRNA, and the mRNA encoding the differentially expressed protein, (b) more indirectly from the interaction between Hfq, sRNA, and a mRNA encoding other regulatory proteins, and (c) downstream effects from these directly and indirectly Hfq‐regulated proteins. When possible, we further identified proteins which translation is directly influenced by Hfq and those that are differentially expressed by indirect regulatory effects. The data were then validated by comparing them with previously published experiments. The resulting identified core set of proteins directly and indirectly regulated by Hfq combined with a review of the *N. meningitidis* metabolome led to a reassessment of the overall function of the Hfq regulon in this obligate human pathogen.

## Materials and methods

### Bacterial strains and culture conditions

The *N. meningitidis* strain H44/76, B:P1.7,16:F3‐3: ST‐32 (cc32), is closely related to the serogroup B strain MC58 and belongs to the same clonal complex 23. *Neisseria meningitidis* H44/76 *hfq* deletion mutant was created as described in a previous article 13. *Neisseria meningitidis* H44/76 was chosen for its high natural competence, it has seen limited plate culture passages since being isolated from a patient in Norway in 1976, and has not been genetically modified 24. *Neisseria meningitidis* H44/76 wild‐type (wt) and H44/76Δ*hfq* (Δ*hfq*) were grown overnight on GC agar plates (Difco, BD Diagnostics, Sparks, MD, USA) supplemented with 1% (v/v) Vitox (Oxoid Ltd, Hampshire, UK) at 37 °C in a humidified atmosphere of 5% CO_2_. Four biological wt replicates and three biological replicates of Δ*hfq* were incubated in 50 mL GC medium supplemented with 1% (v/v) Vitox in 100‐mL Erlenmeyer flasks (OD_530_ ~ 0.05) fixed on a gyratory shaker (180 RPM) at 37 °C. No antibiotic selective pressure was needed since Δ*hfq* was constructed as a complete chromosomal knockout. Previous experiments have shown that in a trans‐complemented mutant the protein expression profile was restored to wt levels 13, therefore this strain was not used in the LC‐MS^E^ experiment. Growth was monitored by measuring optical density of cultures at 530 nm (OD_530_; Pharmacia Biotech Ultraspec 2000, Biochrom Ltd, Cambridge, England) at regular intervals. At the completion of all experiments, cultures were plated on Columbia Agar supplemented with defibrinated sheep blood and incubated at 37 °C to verify cultures were viable and axenic. Cultures were harvested at logarithmic planktonic growth (OD_530_ ~ 0.5, *t* ~ 2 h for wt, *t* ~ 3 h for Δ*hfq*) by pipetting 1 mL (~ 2.5 × 10^9^ CFU) of culture to 1 mL of ice‐cold phosphate‐buffered saline (PBS). The mixture was immediately centrifuged at 16.000 RCF at 4 °C, followed by washing of the pellet with PBS and centrifuged again. Next, pellets were frozen at −20 °C overnight.

### Reverse‐phase liquid chromatography followed by data‐independent alternate scanning mass spectrometry

Frozen pellets were suspended in 0.1% RapiGest SF Surfactant (Waters corporation, Milford, MA, USA)/50 mm NH_4_HCO_3_ pH 8.0 (Sigma‐Aldrich, Darmstadt, Germany), incubated for 1 h on ice and refrozen. Protein content of all samples was determined by standard bicinchoninic acid assay (Thermo Scientific, Rockford, IL, USA) using the manufacturers protocol. Proteolysis of the samples was performed overnight and subsequent removal of Rapigest surfactant was performed according to the protocol provided with Rapigest SF for in solution digestion using 1 : 50 (w/w) ratio of trypsin (Promega, Madison, WI, USA):protein. Next, peptides were mixed 1 : 1 (v/v) with 100 nm ADH1 from *Saccharomyces cerevisiae* digest standard (Waters Corporation, Milford, MA, USA) before being separated by reversed‐phase chromatography and analyzed by data‐independent (MS^E^) label‐free mass spectrometry as described before 25 on a Synapt‐G2 quadrupole time‐of‐flight mass spectrometer (Waters Corporation). Continuum LC‐MS^E^ data were processed and searched using proteinlynx globalserver version 2.5 (PLGS 2.5; Waters). The parameter settings were: digest reagent—trypsin; allow 1 ‘missed cleavage’; search tolerances automatic (typically 5 ppm for precursor and 15 ppm for product ions); fixed modification—cysteine carbamidomethylation; variable modification—methionine oxidation. Protein identifications were obtained by searching *N. meningitidis* MC58 database (UniProt release 2012_03) extended with common protein contaminants, as well as ADH1 from *S. cerevisiae* (the internal standard), to address technical variation and check for concentration differences between samples 20. Details regarding HI3 peptide quantitation can be found in 20 and, especially, 21. Further information regarding reproducibility and reliability with regard to the relative quantitation (wt vs. *hfq* deletion mutant) can be obtained from the supplementary information.

### Data analysis

Differential expression was analyzed by Student's *t*‐test (two‐tailed distribution, equal variances) with false discovery rate (FDR) control according to Benjamini–Hochberg (BH) 26. The original BH procedure was chosen over later approaches that refine for the dependency problem as previous experiments have shown Hfq to regulate a wide range of proteins and the more naïve linear step‐up procedure offers the most conservative estimate in this situation 27. To allow for statistical analysis of proteins only detected in 1 condition, proteins in other samples were assumed to be quantified at least 10% lower than the lowest detected protein (0.11). The samples were given the value 0.10 in order to minimize overestimation of significance and fold‐regulation. Only proteins with *q*‐values ≤ 0.05 FDR (BH) were considered differentially regulated. Gene identification was taken from the original *N. meningitidis* MC58 annotation 28 and updated with the aid of recent literature 29, 30, 31, 32, 33, 34, 35, 36 and BLAST searches 37. Phase and antigenic variable genes were identified as reported 38, 39, 40, 41. A pathway or biological role was given based on KEGG 42 or Uniprot 43. Pathway analysis was further refined to apply specifically to the *N. meningitidis* genome 44, 45, 46, 47, 48, 49, 50, 51. Operon information was derived from previous transcriptome experiments in H44/76 52. Data from previous experiments were taken as reported in table 3 14, table 2 (iron replete condition) 11, table S1 12, and table 2 13.

## Results and Discussion

### Analysis of cellular protein content and reproducibility of LC‐MS^E^ results

Protein concentrations of wt and Δ*hfq* whole‐cell lysates were 1.14 mg·mL^−1^ (SD: 0.08) and 1.29 mg·mL^−1^ (SD: 0.07), respectively. Planktonic cells were harvested during the exponential growth phase at OD_530_ of 0.5 (~ 2.5 × 10^9^ CFU·mL^−1^) and the protein content was estimated to be 0.5 pg per cell for both wt and Δ*hfq* meningococcal strains. Average relative abundance of proteins detected in wt and Δ*hfq* was comparable (R^2^ = 0.82; Fig. S1) and reproducible between individual replicates (Fig. S2). Volcano plots were created to visually represent statistical significance of the largest changes (Fig. S3). Finally, Venn diagrams were created to visualize comparisons of identified proteins between wt and Δ*hfq* strains and within biological replicates of the two strains (Fig. S4). Raw output data from the proteinlynxglobalserver analysis program, encompassing all precursor, fragment, peptide, and protein data extracted from the raw files by the algorithm are available (Data S1). The LC‐MS^E^ analysis in this experiment was shown to be robust and reliable, similar to that described in a recent comparative analysis 21.

### Results of proteomic analysis of wt vs. Δ*hfq*

**Figure 1:**
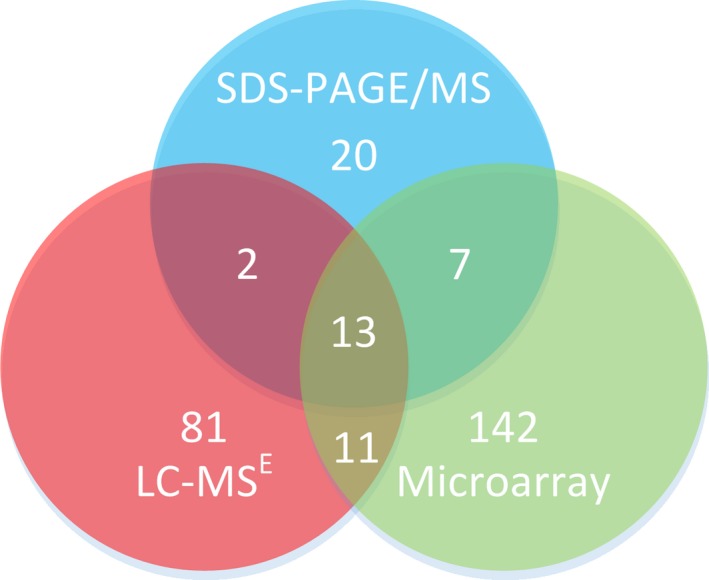
Venn diagram showing correlations between results of this LC‐MS^E^ study in red, the combined SDS/PAGE/MS results of Fantappiè *et al*. 14 and Pannekoek *et al*. 13 in blue, and the combined microarray results of Mellin *et al*. 11 and Fantappiè *et al*. 12 in green.

From 2480 annotated open reading frames in H44/76 23, 937 proteins (38%) were detected. Reliable quantitation of relative expression comparing wt and Δ*hfq* was achieved with 506 proteins (20%). Using FDR control at *q *≤* *0.05, 107 proteins were found differentially expressed, of which 48 and 59 were up‐ and downregulated, respectively (Table S1). From these proteins, 81 were identified as novel Hfq‐regulated candidates, while 15 proteins were previously found by SDS/PAGE/MS and 24 with microarray analyses (Fig. 1). The 107 differentially expressed proteins are involved in a variety of cellular processes and with information derived from the other 399 LC‐MS^E^ detected proteins and previous Hfq analyses a substantial network of metabolic pathways can be constructed (Fig. S5). The major outer membrane proteins (OMPs) PorB and RmpM, which we considered as controls in a nonimmunogenic environment, showed stable expression 17, 53, 54.

**Figure 2:**
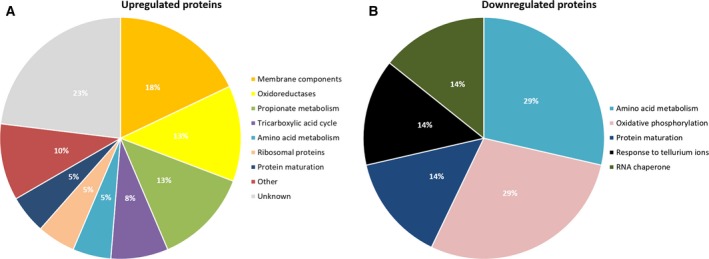
Pie charts depicting the pathway or biological role of (A) upregulated genes and (B) downregulated genes as found in Table 1.

Table 1 was generated to validate the results obtained with LC‐MS^E^ and to create an overview of the genes with robust experimental evidence of regulation by Hfq. It was created by taking all the genes that were consistently differentially regulated in more than one study involving microarray 11, 12, SDS/PAGE/MS 13, 14, and/or LC‐MS^E^ with independently generated isogenic wt vs. Δ*hfq* meningococcal strains. From 107 differentially regulated genes identified by LC‐MS^E^, 27 genes (25%) were independently corroborated by other experiments on the mRNA and/or protein level. Figure 2 is a graphical overview of the pathway or biological role these genes are involved in.

**Table 1:** Summary of all genes consistently differentially regulated between *N. meningitidis* wt vs. Δ*hfq* in more than one study involving independently generated isogenic meningococcal strains

Gene ID^a^	Name^a^	Function^b^	Pathway or Biological role^b^	LC‐MS^E^ c	SDS/PAGE/MS^d^		Microarray^e^	
Upregulated								
NMB0177		Sodium/alanine symporter	Membrane components				13.1	4.0
NMB0227		Mn^2+^–iron transporter	Membrane components				3.3	2.4
NMB0317	*queF*	7‐cyano‐7‐deazaguanine reductase	tRNA modification	4.5				4.5
NMB0325	*rplU*	50S ribosomal protein L21	Ribosomal proteins	22.9				5.2
NMB0430	*prpB*	2‐methylisocitrate lyase	Propionate metabolism	9.1		↑	20.4	28.2
NMB0431	*prpC*	Methylcitrate synthase	Propionate metabolism	3.8	1.6	↑	23.9	56.7
NMB0432	*tuaE* f	Anion (sulfite) transporter	Membrane components & Propionate metabolism				5.9	3.9
NMB0435	*ackA‐1*	Acetate kinase	Propionate metabolism	14.9	2.0		13.7	18.1
NMB0546	*adhP*	Alcohol dehydrogenase	Oxidoreductases	1.5		↑	1.7	
NMB0574	*gcvT*	Aminomethyltransferase	Amino acid metabolism	3.1				2.6
NMB0589	*rplS*	50S ribosomal protein L19	Ribosomal proteins	3.7		↑		7.3
NMB0634	*fpbA*	Iron ABC transporter	Membrane components	1.3	1.5	↑		
NMB0649		Hypothetical protein	Unknown				2.8	5.3
NMB0650		Hypothetical protein	Unknown	9.4				4.8
NMB0791	*ppiB*	Peptidyl–prolyl *cis–trans* isomerase B	Protein folding	2.1		↑		2.4
NMB0884	*sodB*	Superoxide dismutase, Fe–Mn	Oxidoreductases	3.5	1.7	↑		
NMB0859		Hypothetical protein	Unknown				3.2	2.7
NMB0861		Hypothetical protein	Unknown				2.1	2.8
NMB0865		Hypothetical protein	Membrane components				5.9	12.8
NMB0866		Hypothetical protein	Unknown				4.8	8.9
NMB0920	*icd*	Isocitrate dehydrogenase	TCA cycle	5.8	1.4	↑		5.0
NMB0946	*prx*	Peroxiredoxin 2 protein/glutaredoxin	Oxidoreductases		1.4	↑		2.0
NMB0954	*gltA*	Citrate synthase	TCA cycle	5.9	2.0	↑		4.4
NMB1055	*glyA*	Serine hydroxymethyltransferase	Amino acid metabolism	6.5	3.0			5.4
NMB1306	*zapE*	ATPase	Cell division	2.6				2.8
NMB1378	*iscR* f	Iron–sulfur cluster assembly transcription factor	Iron–sulfur cluster biosynthesis	6.1				2.1
NMB1388	*pgi‐1*	Glucose‐6‐phosphate isomerase 1	Glycolysis/Gluconeogenesis	11.1	1.3	↑		2.7
NMB1398	*sodC*	Superoxide dismutase, Cu‐Zn	Oxidoreductases			↑		2.3
NMB1406		Hypothetical protein	Membrane components				3.4	3.6
NMB1572	*acnB*	Aconitate hydratase 2	TCA cycle & Propionate metabolism	7.6	2.3	↑	2.7	4.5
NMB1584	*mmsB* f	3‐hydroxyacid dehydrogenase	Unknown	38.9	1.9	↑		6.7
NMB1599		Hypothetical protein	Unknown				17.0	6.8
NMB1600		Hypothetical protein	Unknown				3.8	3.6
NMB1764		Hypothetical protein	Unknown				3.4	2.9
NMB1796		FMN reductase	Oxidoreductases	2.6	2.9	↑		3.3
NMB1946	*metQ* f	Lipoprotein NlpA family	Membrane components	2.3				2.6
NMB2136		Oligopeptide transporter	Protein transport/translocation				3.7	4.0
Downregulated								
NMB0335	*dapD*	Tetrahydropyridine‐carboxylate succinyltransferase	Amino acid metabolism	−1.5	−2.3			
NMB0378	*cysP* f	Inorganic phosphate transporter	Membrane components	−7.7			−1.7	
NMB0543	*lctP* f	Putative l‐lactate permease	Membrane components				−2.2	−3.5
NMB0607	*secD*	Protein translocase subunit	Protein transport/translocation	−4.0			−2.0	−2.6
NMB0748	*hfq*	Host factor‐I protein	RNA chaperone				−289.3^g^	−16.0^g^
NMB0763	*cysK*	Cysteine synthase	Amino acid metabolism		−2.4		−2.7	
NMB0881	*cysT* f	Sulfate transport system permease	Membrane components				−7.3	−2.2
NMB1617	*tehB*	Tellurite resistance protein/methyltransferase	Response to tellurium ion				−3.0	−2.1
NMB1934	*atpD*	ATP synthase subunit beta	Oxidative phosphorylation	−1.7		↓	−1.8	−2.2
NMB1935	*atpG*	ATP synthase gamma chain	Oxidative phosphorylation	−1.8				−2.4

^a^ Gene identification and name according to Tettelin *et al*. 28, updated based on literature published since. ^b^ Function, pathway, or biological role according to the Kyoto Encyclopedia of Genes and Genomes (http://www.kegg.jp/) and/or UniProt (http://www.uniprot.org/). ^c^ LC‐MS^E^ results from this study, all genes *q *≤* *0.05 except NMB0791 (*P *=* *0.026). ^d^ SDS/PAGE/MS results taken from Fantappiè *et al*. 14 and Pannekoek *et al*. 13, respectively. ^e^ Microarray results taken from Mellin *et al*. 11 and Fantappiè *et al*. 12, respectively. ^f^ Inferred from homology with annotated genes in different strains or species. ^g^ This concerns the ratio of the signal transcripts from *hfq* in the wt and Δ*hfq*. See Materials and methods for references.

Four genes that were found to be upregulated in previous experiments did not reach *q *≤* *0.05 in our LC‐MS^E^ analysis; *ppiB* (2.1 fold, *P *=* *0.026), *fbpA* (1.3 fold, *P *=* *0.058), *prx* (1.2 fold, *P *=* *0.39), and NMB0865 (1.8 fold, *P *=* *0.44). Similarly, *cysK* was found to be significantly downregulated in two previous independent SDS/PAGE/MS and microarray experiments and was stably expressed at *P *=* *0.48 in our LC‐MS^E^ dataset (Table S1). The wt expression of *ppiB*,* fbpA*, and *prx* showed a relatively high variation that resulted in an insignificant statistical level. The conflicting results of NMB0865 and *cysK* could be explained by regulatory differences between *N. meningitidis* strains H44/76 and MC58. Five antigenic or phase variable genes (*fixP*,* pilE*,* pilS*,* omp85*,* tspA*) were found to be inversely regulated when comparing LC‐MS^E^ results with those from the other experiments. Finally, NMB2091 which is included in the novel 4CMenB vaccine 29, is downregulated in our LC‐MS^E^ experiment while previously it was detected as upregulated in our laboratory using SDS/PAGE/MS utilizing the same strain 13. This might suggest variable adaptions to altered OMP assembly in the Δ*hfq* strain as described below.

### Validity of LC‐MS^E^ analysis in analyzing the Hfq regulon in *N. meningitidis*

LC‐MS^E^ is a proteomic technique that has been used successfully in a variety of experiments where absolute quantitation of a complex mix of proteins was desired 21. Based on our results, it has shown to be highly reproducible and has extensively expanded the Hfq regulon in *N. meningitidis* on the level that is ultimately the most relevant for post‐transcriptional regulation, that is, the protein level.

A low correlation between proteomic and transcriptomic data has been reported in several studies, including those involving pathogenic *Neisseria* spp. 17, 18, 55. However, 13 of the top 15 upregulated proteins (87%) detected by LC‐MS^E^ were corroborated independently by analyses performed by other groups. The remaining two proteins that were detected by LC‐MS^E^ but not microarray analysis will be further discussed below. One protein, PrpF, for which no mRNA transcript was detected by microarray is most likely regulated in convergence with the other proteins in the large genomic island responsible for utilization of propionic acid 48. These are among the highest upregulated genes in both LC‐MS^E^ and microarray experiments. The other protein, NMB1381 (annotated as the iron‐binding protein IscA in other meningococcal genomes) has a role in iron–sulfur cluster biogenesis and is part of a group of related proteins that are significantly upregulated (see below). One of the major modes of activity of Hfq in conjunction with sRNAs is the inhibition of translation by sequestering the ribosome‐binding site (RBS) of mRNAs 7. mRNAs that are prevented from entry by the ribosome but not (completely) degraded may still be detected using oligomer microarray approaches (and short read length transcriptome sequencing) but protein products will be absent and therefore not detected by proteomic techniques like LC‐MS^E^.

Intriguingly, the majority of the downregulated proteins detected by LC‐MS^E^ were not detected by microarray. Using LC‐MS^E^, most of these proteins were detected in three or more biological replicates of the wt condition but not in any of the three biological replicates of the *hfq* deletion mutant. We have chosen a conservative approach for detecting downregulated proteins by setting all nondetected proteins just below the detection limit of 0.11 and a rigorous FDR control with result that most of the downregulated proteins were not significantly different in expression between the wt and the *hfq* deletion mutant. There were no discrepancies between those downregulated genes detected in the two microarray experiments and those detected by LC‐MS^E^.

### Comparison with previously characterized sRNAs in *N. meningitidis*

Currently, only a limited number from a myriad of detected putative sRNAs in *N. meningitidis* have been further characterized. A small antisense RNA (AS RNA) *cis*‐encoded on the *pilE* locus modulates pilin variation 56. Its Hfq dependence is unclear, in our Δ*hfq* strain *pilE* expression is highly variable, quantified at the same or at almost half of wt levels. This is in contrast to the increased expression seen in MC58 12, 14 but may be explained by phase variation in this locus encoding antigenically variable proteins. The ferric uptake regulator (Fur)‐regulated sRNA NrrF downregulates the *sdhCDAB* regulon during iron starvation 11, 15, 57. This regulation has been shown to be independent of Hfq and accordingly *sdhA* was found to be stably expressed in our LC‐MS^E^ data. The FNR regulated and anaerobically induced sRNA AniS has been shown to downregulate the genes NMB0214 (encoding PrlC, an oligopeptidase A) and NMB1468 (encoding an immunogenic surface exposed lipoprotein 30) in an Hfq‐dependent fashion 12. Ultimate validation of direct targeting of NMB1468 by AniS was shown using a green fluorescent protein‐based plasmid system in a heterologous *Escherichia coli* background 58, 59. Neither gene was detected by microarray or SDS/PAGE/MS screens of Hfq‐regulated proteins. Furthermore, as NMB1468 was not detected in several proteomic experiments in *N. meningitidis*, it was considered to be present at low levels or inefficiently extracted 30. In our LC‐MS^E^ data, NMB0214 was significantly upregulated 1.7‐fold (*q *=* *0.0031) and NMB1468 was found to be highly expressed and upregulated 1.4‐fold (*P *=* *0.026). This highlights the sensitivity of LC‐MS^E^ to detect subtle but biologically relevant differential regulation even in proteins that are difficult to detect by traditional SDS/PAGE/MS. Finally, sRNA Bns1 was detected in *N. meningitidis* strain MC58 from *ex vivo* glucose‐rich human blood 60 and was subsequently confirmed to be glucose inducible 47. Indirect proof that Bns1 positively regulates NMB0429, which is part of the NMB0432‐PrpB‐PrpC operon, was provided by microarray analysis and *in silico* target prediction, possibly by stabilizing their mRNAs 61. Indeed, a MC58Δ*bns1* strain shows downregulation of this operon. The *prp* gene cluster NMB0430‐NMB0435 was identified as a large genomic island allowing the meningococcus to utilize propionic acid 48. In our study, PrpB, PrpC, PrpF, AckA‐1, and NMB0432 are highly upregulated in the Δ*hfq* background. These results seem to be in contrast with the results obtained with the MC58Δ*bns1* strain. The involvement of Hfq in the upregulation of the NMB0429‐NMB0430 operon, which still needs experimental confirmation, might be complex.

### The Hfq regulon of *N. meningitidis*

Hfq has been studied extensively, particularly in *Enterobacteriaceae* 6, 7, 10, 62. Its central role in facilitating metabolic and structural adaptations in response to environmental factors results in dramatically altered phenotypes in deletion mutants. The alterations observed with LC‐MS^E^ proteomics in the *hfq* knockout vs. the wt reflect the attempt of the bacterium to adapt and grow utilizing pathways and available metabolites. In conjunction with its cellular nucleotide and protein partners, Hfq allows for both up‐ and downregulating post‐transcriptional effects. The resulting proteome will reflect the highly complex effects of abrogating Hfq. These effects comprise both the result of direct interaction between Hfq and its sRNA and mRNA targets and the indirect effects that Hfq might have in interaction with sRNAs targeting mRNAs that code for regulators. However, several consistent trends can be discovered in the current and previously reported experiments that will be discussed in the following paragraphs.

As shown before 13, the *hfq* deletion mutant of *N. meningitidis* demonstrates a severely hampered growth rate. The results of this proteomic study provide a plausible explanation showing highly downregulated genes involved in nucleotide synthesis, DNA replication, cell division, lipopolysaccharides (LPS), and peptidoglycan synthesis, membrane components, protein biosynthesis, protein folding, amino acid metabolism, fatty acid metabolism, and cofactor and vitamin metabolism. Furthermore, many proteins involved in releasing energy using oxidative phosphorylation (ATP synthesis coupled with the electron transport chain) are downregulated.

The growth retardation and downregulation of structural proteins can be the consequence of the reduced ability of the meningococcus to respire and generate energy. Hfq may also be directly associated with the synthesis of, for example, membrane components, which has been speculated for LpxD (involved in LPS lipid‐A synthesis) 10. In *E. coli* the deletion of *hfq* causes the activation of the σ^E^ and Cpx cell envelope stress responses that are caused by deregulation of OMPs 63. Several OMPs and their associated proteins involved in biogenesis and folding, are downregulated in Δ*hfq* (e.g., ComL, fHbp, GNA2091/YrAP 29, SurA, and FkpA) while others are stably expressed (PorB and RmpM). This suggests specific Hfq‐associated regulation of outer membrane biogenesis in *N. meningitidis*. The σ^E^‐regulon in *N. meningitidis* is surprisingly small 52 and a homolog of Cpx is lacking, contrary to *E. coli*. This represents an example where Hfq regulation in *N. meningitidis* and the gram‐negative model organism *E. coli* are similar but different.

Interestingly, in Δ*hfq* six of the seven proteins involved in propionate metabolism ending in succinate, pyruvate, and oxaloacetate (NMB0432, AckA‐1, PrpC, PrpF, AcnB, and PrpB) and four of the five proteins involved in the tricarboxylic acid (TCA) cycle converting malate to α‐ketoglutarate (YojH, GltA, AcnB, and Icd) are among the highest upregulated genes. Crucially, aconitate hydratase B (AcnB) plays a dual role in catalyzing both the reaction 2‐methyl‐*cis*‐aconitate ↔ 2‐methylisocitrate of the methylcitrate cycle and the reaction *cis*‐aconitate ↔ isocitrate of the TCA cycle. Proteins involved in the Entner–Doudoroff pathway, the preferred glucose breakdown route in *N. meningitidis*, show downregulation (*P *≤* *0.05) indicating a shift away from glucose catabolism 64. The lactate permease LctP that transports extracellular lactate into the cytosol is downregulated, impairing the use of lactate as a carbon source. This has profound effects on the ability of *N. meningitidis* to grow both *in vitro* 65 and *in vivo* 49, 66, 67, and in colonizing the nasopharynx 68 and immune evasion 69, 70.

In the pentose phosphate pathway, only glucose‐6‐phosphate dehydrogenase encoded by *zwf* is significantly downregulated and together with Glp catalyze the unidirectional oxidation of glucose 6‐phosphate (G6P) to 6‐phosphogluconolactone (6PG). Zwf shares a similar evolutionary origin and enzymatic mechanism with the 3‐hydroxyisobutyrate dehydrogenase MmsB as they are part of the 3‐hydroxyacid dehydrogenase family 71. NMB1584 in *N. meningitidis* encodes a putative 3‐hydroxyisobutyrate dehydrogenase similar to MmsB and shows the highest upregulated fold change in our LC‐MS^E^ experiment. Its function in the meningococcus is not characterized but in other bacteria MmsB has been shown to generate energy by catabolizing amino acids 72. This suggests the existence of an Hfq‐dependent mechanism influencing NMB1584 mRNA expression and regulating catabolism of amino acids for alternative energy when needed in nutrient‐poor environments.

In the partially functioning Embden Meyerhof Parnas pathway fructose bisphosphatase (fbp) is highly downregulated while glycolytic glucose‐6‐phosphate isomerase pgi‐1 is highly upregulated. In the gram‐negative model organisms *E. coli* and *Salmonella enterica*, intracellular glucose levels are strictly controlled and glucose homeostasis is subject to complex transcriptional and post‐transcriptional control 73. Examples include the prevention of phosphosugar stress and the control of amino sugar biosynthesis 74. Genes involved in these pathways show dramatic alterations upon the deletion of *hfq* from the *Neisserial* chromosome.

The utilization of propionic acid in the adult nasopharynx has been proposed to provide the meningococcus with a selective advantage. In this ecological niche many anaerobes produce propionate as the end‐product of fermentation. This provides a carbon source the meningococcus can use for growth 48. The end products of the methyl‐citrate cycle, succinate, pyruvate, and oxaloacetate, feed directly into the TCA cycle where the oxidation of acetyl‐CoA is highly upregulated. The active import of extracellular propionate and activation of the methyl‐citrate cycle are either the consequences of deregulation of the genes involved in this pathway (e.g., through Bns1 or an undiscovered other sRNA) or the more indirect result of the meningococcus opting for propionic acid as a carbon source following the inability to utilize other carbon sources. Furthermore, the upregulation of genes involved in a specific part of the TCA cycle is striking and perhaps indicative of a direct effect of the loss of Hfq‐dependent function of a sRNA.

The highly upregulated genes involved in propionic acid metabolism and the TCA cycle causes high levels of α‐ketoglutarate resulting in a dampening effect on GdhA (−1.8 fold, *P *=* *0.014) 75, which is involved in α‐ketoglutarate l‐glutamate interconversion. This could explain the strongly downregulated genes involved in the import and biosynthesis of l‐glutamate (*gltT* and *putA*, respectively), as l‐glutamate is not siphoned off into the TCA cycle by GdhA. Its conversion into glutathione, however, is limited by the availability of cysteine (see below).

Other clusters of upregulated genes are those involved in iron–sulfur biosynthesis (*iscR*,* iscA*,* iscS*,* fdx‐1*/*2*,* nifU*/*iscU*,* cyaY*) and iron storage (*bfrA* and *bfrB*). The genes coupled with the upregulation of oxidoreductases SodB and SodC resemble a response of the meningococcus to abundant iron conditions and oxidative stress. Surprisingly in this context, the proteins CysK and CysT which are involved in acquiring extracellular H_2_S and SO_4_^2−^ required for cysteine synthesis, are downregulated. This might lead to cysteine depletion which causes oxidative stress and impairs iron–sulfur protein assembly 76.

The sRNA RyhB in *E. coli* has been shown to regulate *sodB* and the *iscRSUA* polycistronic mRNA by targeting it for degradation by RNase E under iron limitation conditions 77. Genes that are upregulated in iron‐deprived conditions, including Fur, are stably expressed in the absence of Hfq when the bacterium is growing with abundant iron 17, 18, 78. It has been proposed that in *N. meningitidis*, similar to other bacteria, intracellular iron homeostasis is the target of tight post‐transcription control 77. To date, the only sRNA found to be regulated by Fur in *N. meningitidis* is NrrF 57. Its regulating abilities has been found to be limited thus far, leaving room for additional sRNA regulators to be involved in iron homeostasis in this bacterium.

*Neisseria meningitidis* expresses a Zinc uptake regulator (Zur) that represses proteins involved in zinc uptake such as ZnuD 79. NMB0546 (the zinc‐containing alcohol dehydrogenase AdhP) is known to be downregulated under conditions of zinc limitation, while NMB0317 (NADPH‐dependent 7‐cyano‐7‐deazaguanine reductase QueF, involved in queuosine biosynthesis) is downregulated when zinc is abundant. Both genes are upregulated in the *hfq* deletion mutant strain. Therefore, the interplay of Hfq, Zur, and possible sRNA intermediates might have activating and repressing effects.

Bacterial ribosomes are traditionally seen as homogeneous entities that consist of the same set of ribosomal proteins and rRNA molecules to accomplish protein synthesis. However, evidence for subpopulations of heterogeneous and functionally specialized ribosomes that react to environmental stimuli has emerged 80. Furthermore, a large body of *cis*‐ and *trans*‐oriented noncoding RNA candidates associated with ribosomal protein operons have been identified, of which several *trans*‐acting sRNAs were differentially regulated by Hfq 81. In our study, five ribosomal proteins and the putative 23S rRNA methyltransferase NMB0475 were upregulated while another five ribosomal proteins and the ribosomal maturation factor RimP are downregulated. Interestingly, all five ribosomal proteins that are upregulated and one of the downregulated ribosomal proteins have been shown to interact with Hfq in *E. coli* 10, 82. As the majority of proteins of fully assembled ribosomes are readily exchangeable 83 the specialization of the translational machinery could potentially be completely modulated by ribo‐regulation facilitated by Hfq.

## Conclusions

The analysis and validation of the Hfq regulon in *N. meningitidis* has given further insight into its profound regulatory effects. In conjunction with previous studies, a comprehensive network of Hfq‐regulated proteins was constructed and differentially expressed proteins were found to be involved in a large variety of cellular processes. Potential gaps in the Hfq‐dependent sRNA repertoire in the meningococcus were identified in either the direct or indirect regulation of OMPs, the methyl‐citrate and TCA cycles, iron and zinc homeostasis, and the assembly of ribosomal proteins. Possible analogs in more canonical gram‐negative *Enterobacteriaceae* have been described but further research is needed to identify and characterize these sRNAs in *N. meningitidis*.

## Data accessibility

Raw output data from the ProteinLynxGlobalServer analysis program, encompassing all precursor, fragment, peptide, and protein data extracted from the raw files by the algorithm. This data is located at figshare.com: https://dx.doi.org/10.6084/m9.figshare.5001854 [Correction added after online publication on 5 June 2017: figshare data information updated].

## Author contributions

RHV, AvdE, DS, and YP conceived and designed the project. RHV and GK acquired the data. RHV, GK, AvdE, DS, and YP analyzed and interpreted the data. RHV wrote the original draft and GK, AvdE, DS, and YP contributed to the final manuscript.

## Supporting information


**Fig. S1.** Relative abundance of proteins of wt and *hfq* deletion mutant strains.

**Fig. S2.** Replicate analysis plots.

**Fig. S3.** Volcano plots.

**Fig. S4.** Protein comparisons between and within biological replicates of wt and *hfq* deletion mutant strains.

**Fig. S5.** Schematic representation of metabolic pathways influenced by Hfq.

**Table S1.** Extended results of LC‐MS^E^ experiments.

**Data S1**. Supplementary materials.
