# Supplementary figures and images for: The Hfq regulon of Neisseria meningitidis
Source: FEBS Open Bio. 2017 Apr 25;7(6):777–88. doi: 10.1002/2211-5463.12218 (PMC5458458; doi:10.1002/2211-5463.12218)

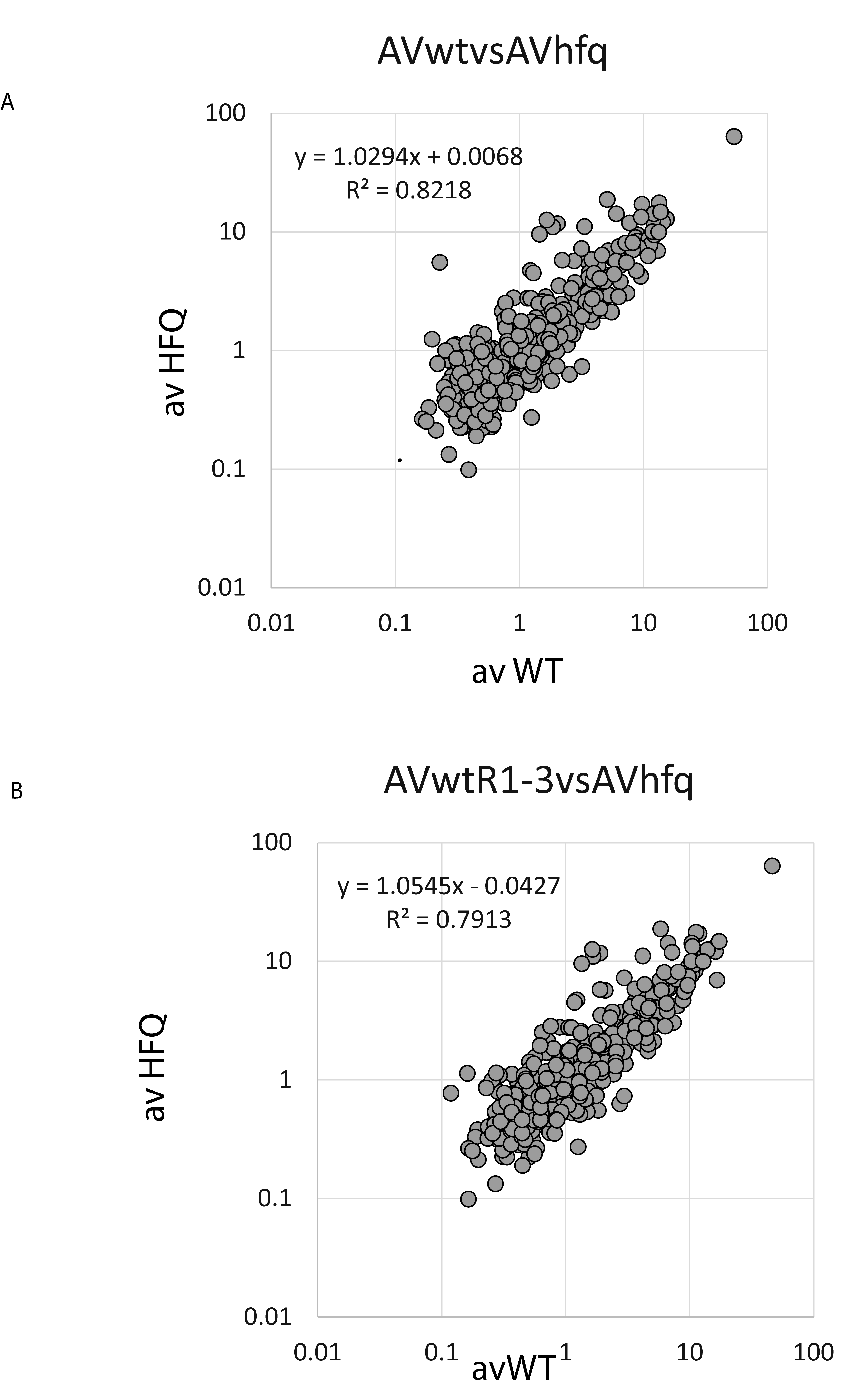

Supplement: Supplementary file 1 — Fig. S1. Relative abundance of proteins of wt and hfq deletion mutant strains. [file FEB4-7-777-s001.tif]

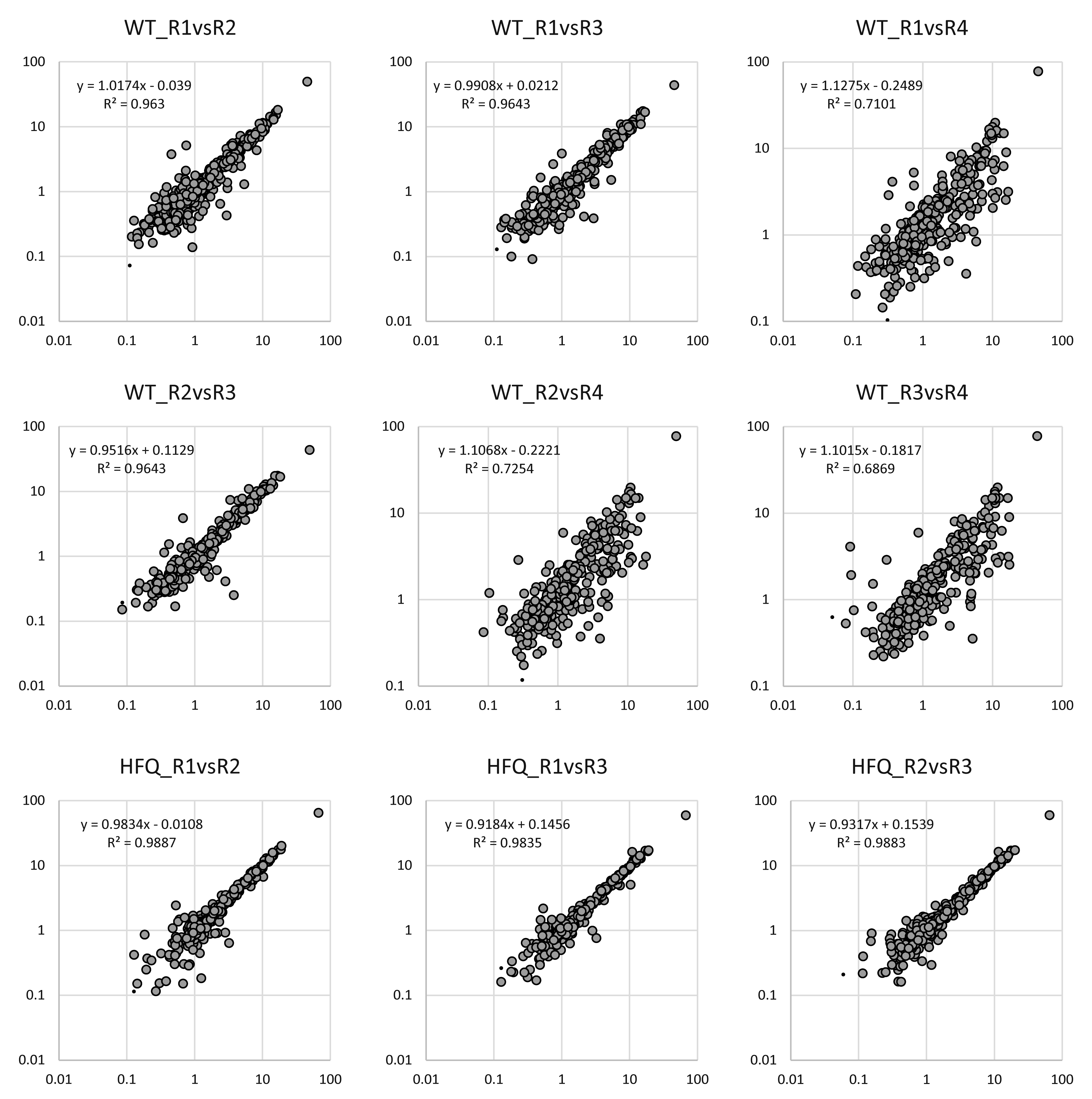

Supplement: Supplementary file 2 — Fig. S2. Replicate analysis plots. [file FEB4-7-777-s002.tif]

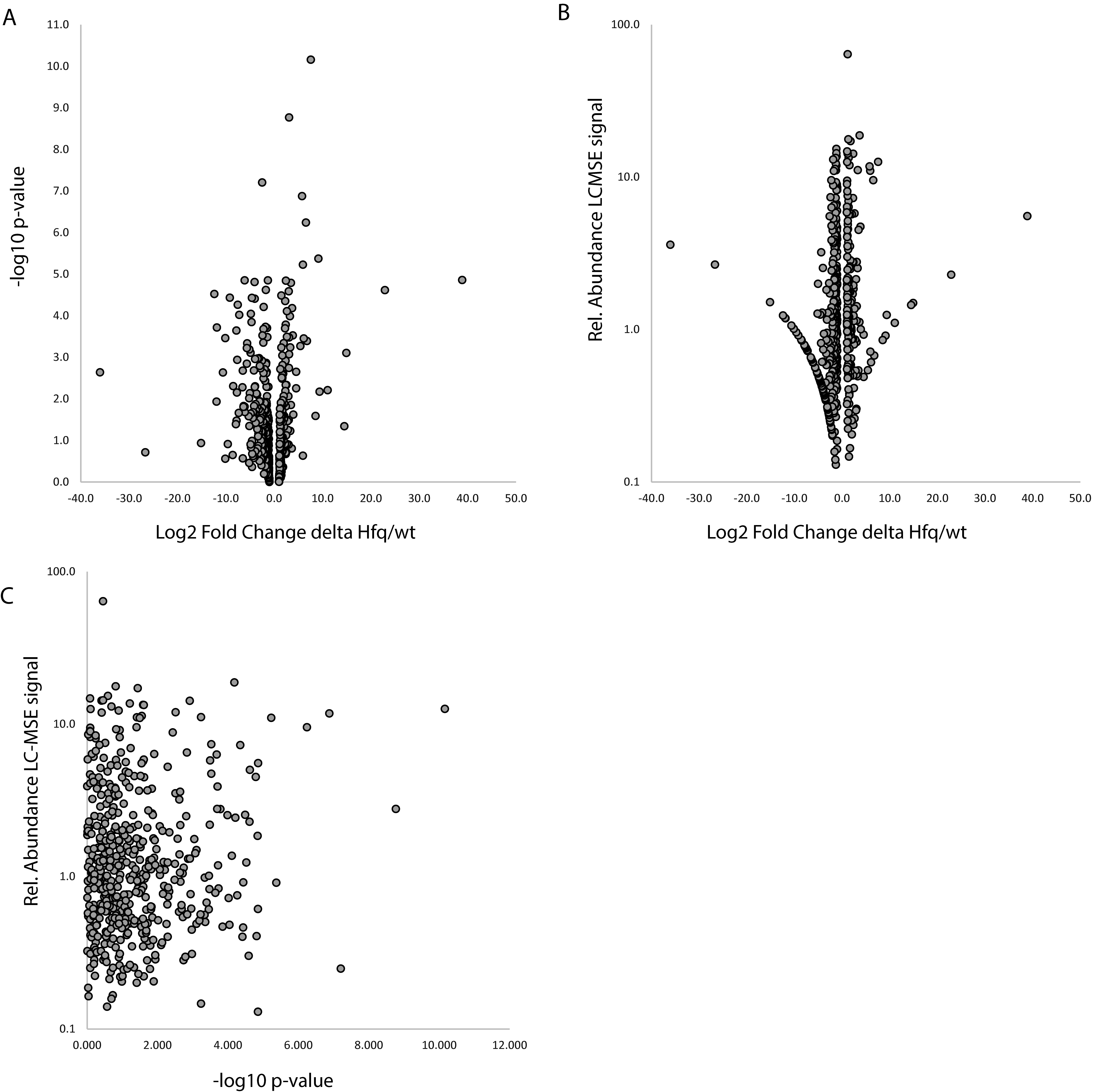

Supplement: Supplementary file 3 — Fig. S3. Volcano plots. [file FEB4-7-777-s003.tif]

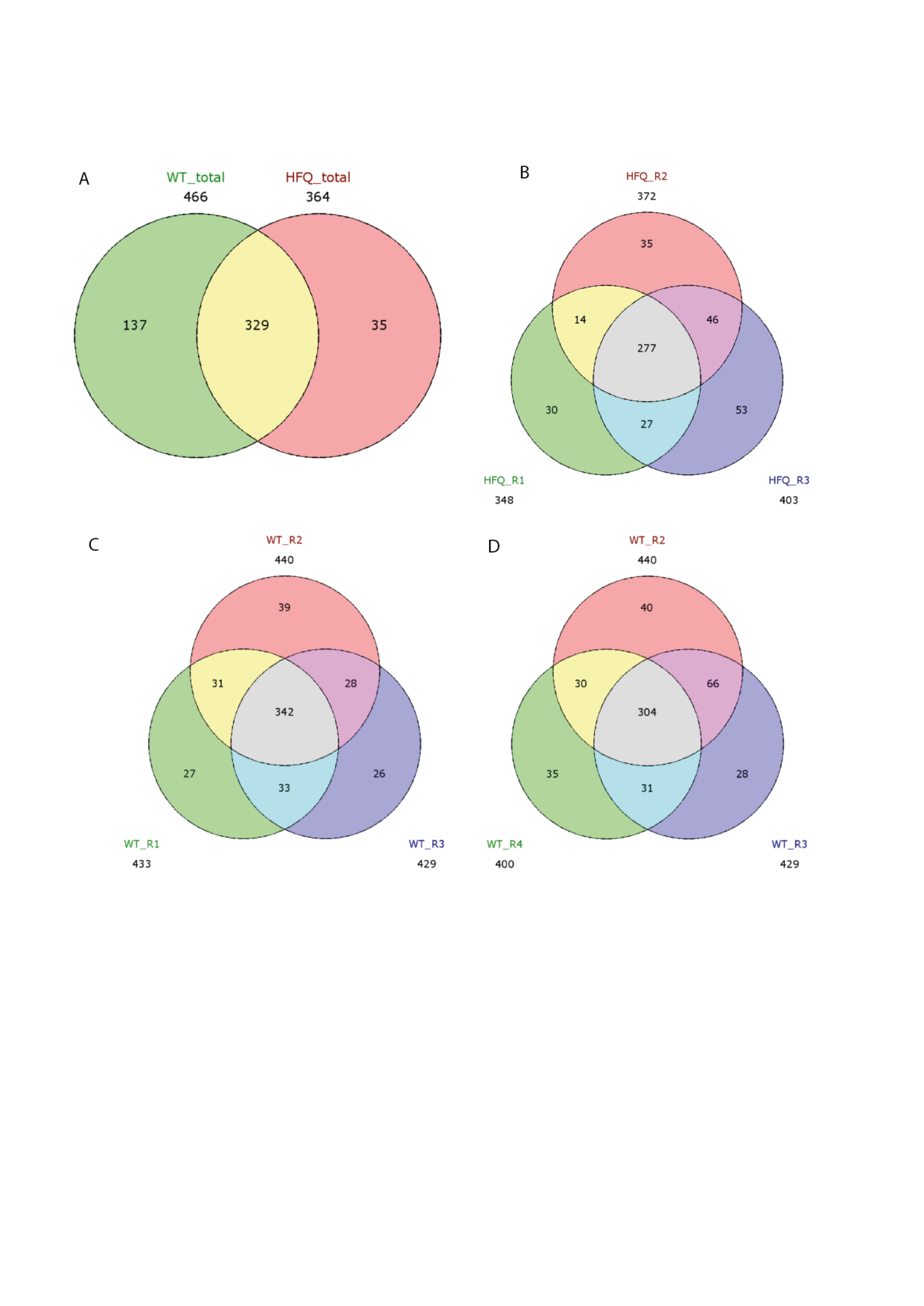

Supplement: Supplementary file 4 — Fig. S4. Protein comparisons between and within biological replicates of wt and hfq deletion mutant strains. [file FEB4-7-777-s004.tif]
