# Supplementary material for: The Hfq regulon of Neisseria meningitidis
Source: FEBS Open Bio. 2017 Apr 25;7(6):777–88. doi: 10.1002/2211-5463.12218 (PMC5458458; doi:10.1002/2211-5463.12218)
Supplement: Supplementary file 5 — Fig. S5. Schematic representation of metabolic pathways influenced by Hfq. [file FEB4-7-777-s005.pdf]

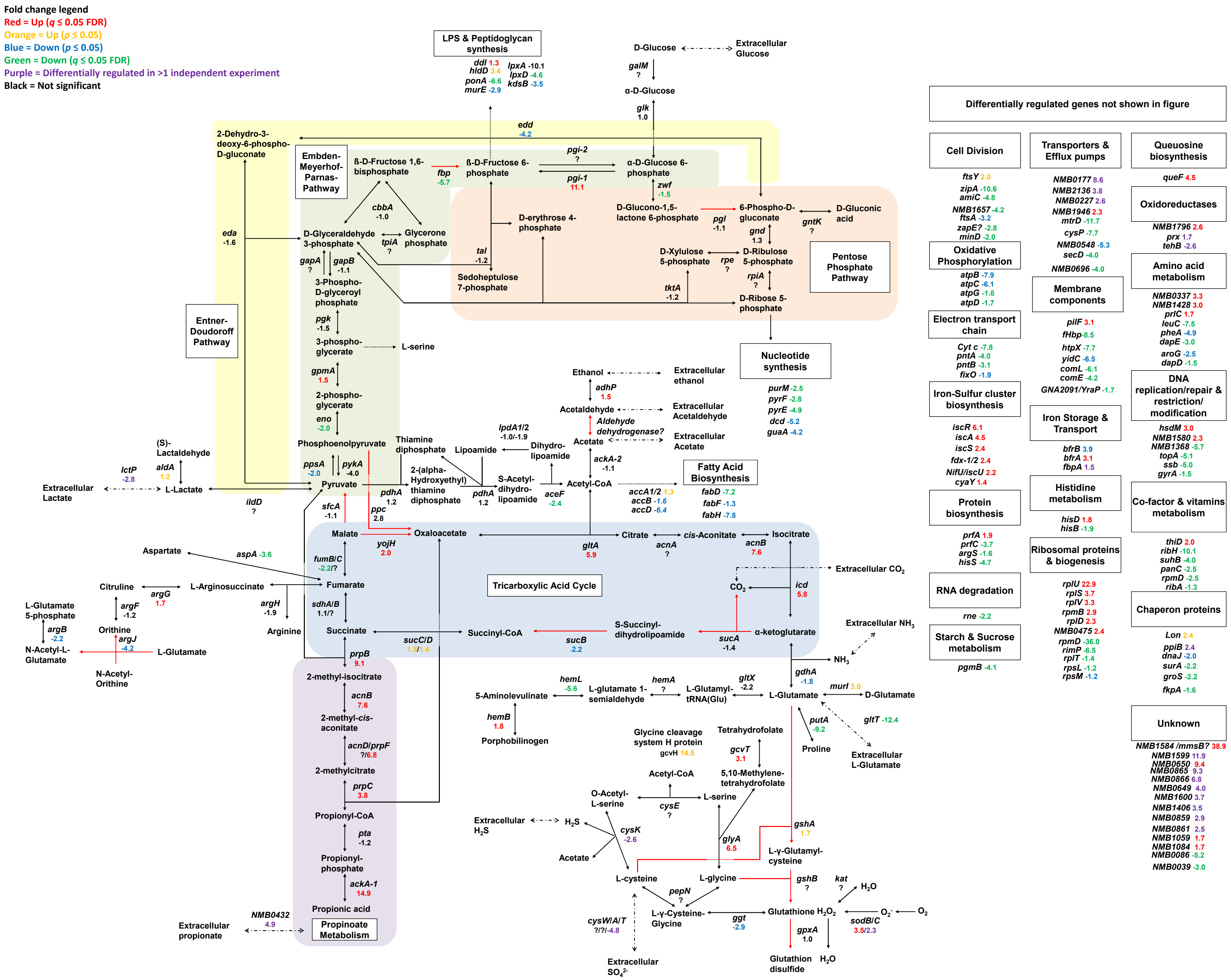

Fold change legend
Red = Up (q ≤ 0.05 FDR)
Orange = Up (p ≤ 0.05)
Blue = Down (p ≤ 0.05)
Green = Down (q ≤ 0.05 FDR)
Purple = Differentially regulated in >1 independent experiment
Black = Not significant
LPS & Peptidoglycan synthesis
ddl 1.3
hldD 3.4
ponA -6.6
murE -2.9
lpxA -10.1
lpxD -4.6
kdsB -3.5
D-Glucose
Extracellular Glucose
galM ?
α-D-Glucose
glk 1.0
edd -4.2
2-Dehydro-3-deoxy-6-phospho-D-gluconate
Embden-Meyerhof-Parnas-Pathway
ß-D-Fructose 1,6-bisphosphate
fbp -5.7
ß-D-Fructose 6-phosphate
pgi-2 ?
pgi-1 11.1
α-D-Glucose 6-phosphate
zwf -1.5
D-Glucono-1,5-lactone 6-phosphate
pgl -1.1
6-Phospho-D-gluconate
gntK ?
D-Gluconic acid
gnd 1.3
D-Ribulose 5-phosphate
D-Xylulose 5-phosphate
rpe ?
rpiA ?
D-Ribose 5-phosphate
tktA -1.2
Pentose Phosphate Pathway
D-erythrose 4-phosphate
Sedoheptulose 7-phosphate
tal -1.2
cbbA -1.0
eda -1.6
D-Glyceraldehyde 3-phosphate
tpiA ?
Glycerone phosphate
gapA ?
gapB -1.1
3-Phospho-D-glyceroyl phosphate
pgk -1.5
3-phospho-glycerate
L-serine
gpmA 1.5
2-phospho-glycerate
eno -2.0
Phosphoenolpyruvate
Entner-Doudoroff Pathway
ppsA -2.0
pykA -4.0
Pyruvate
(S)-Lactaldehyde
aldA 1.2
lctP -2.8
Extracellular Lactate
L-Lactate
lldD ?
sfcA -1.1
ppc 2.8
Thiamine diphosphate
pdhA 1.2
2-(alpha-Hydroxyethyl) thiamine diphosphate
Lipoamide
lpdA1/2 -1.0/-1.9
Dihydro-lipoamide
pdhA 1.2
S-Acetyl-dihydro-lipoamide
aceF -2.4
Acetyl-CoA
Ethanol
Extracellular ethanol
adhP 1.5
Acetaldehyde
Extracellular Acetaldehyde
Aldehyde dehydrogenase?
Acetate
Extracellular Acetate
ackA-2 -1.1
Fatty Acid Biosynthesis
accA1/2 1.3
accB -1.6
accD -6.4
fabD -7.2
fabF -1.3
fabH -7.8
Nucleotide synthesis
purM -2.5
pyrF -2.8
pyrE -4.9
dcd -5.2
guaA -4.2
Malate
yojH 2.0
Oxaloacetate
gltA 5.9
Citrate
acnA ?
cis-Aconitate
acnB 7.6
Isocitrate
Extracellular CO2
CO2
icd 5.8
Tricarboxylic Acid Cycle
fumB/C -2.2/?
Fumarate
Aspartate
aspA -3.6
Citruline
argG 1.7
L-Arginosuccinate
argF -1.2
argH -1.9
Arginine
L-Glutamate 5-phosphate
argB -2.2
Orinithine
argJ -4.2
N-Acetyl-L-Glutamate
L-Glutamate
N-Acetyl-Orithine
sdhA/B 1.1/?
Succinate
sucC/D 1.8/1.4
Succinyl-CoA
sucB -2.2
S-Succinyl-dihydrolipoamide
sucA -1.4
α-ketoglutarate
Extracellular NH3
NH3
gdhA -1.8
prpB 9.1
2-methyl-isocitrate
acnB 7.6
2-methyl-cis-aconitate
acnD/prpF ?/6.8
2-methylcitrate
prpC 3.8
Propionyl-CoA
pta -1.2
Propionyl-phosphate
ackA-1 14.9
Propionic acid
Propinoate Metabolism
NMB0432 4.9
Extracellular propionate
5-Aminolevulinate
hemL -5.6
L-glutamate 1-semialdehyde
hemA ?
L-Glutamyl-tRNA(Glu)
gltX -2.2
L-Glutamate
murI 3.0
D-Glutamate
hemB 1.8
Porphobilinogen
putA -9.2
Proline
gltT -12.4
Extracellular L-Glutamate
Glycine cleavage system H protein
gcvH 14.5
Tetrahydrofolate
gcvT 3.1
5,10-Methylene-tetrahydrofolate
Acetyl-CoA
O-Acetyl-L-serine
cysE ?
L-serine
Extracellular H2S
H2S
cysK -2.6
Acetate
glyA 6.5
gshA 1.7
L-γ-Glutamyl-cysteine
L-cysteine
L-glycine
gshB ?
pepN ?
L-γ-Cysteine-Glycine
ggt -2.9
Glutathione
H2O2
kat ?
H2O
O2-
O2
sodB/C 3.5/2.3
gpxA 1.0
Glutathion disulfide
H2O
cysW/A/T ?/?/-4.8
Extracellular SO4 2-
Differentially regulated genes not shown in figure
Cell Division
ftsY 2.0
zipA -10.6
amiC -4.8
NMB1657 -4.2
ftsA -3.2
zapE? -2.8
minD -2.0
Oxidative Phosphorylation
atpB -7.9
atpC -6.1
atpG -1.8
atpD -1.7
Electron transport chain
Cyt c -7.8
pntA -4.0
pntB -3.1
fixO -1.9
Iron-Sulfur cluster biosynthesis
iscR 6.1
iscA 4.5
iscS 2.4
fdx-1/2 2.4
NifU/iscU 2.2
cyaY 1.4
Protein biosynthesis
prfA 1.9
prfC -3.7
argS -1.6
hisS -4.7
RNA degradation
rne -2.2
Starch & Sucrose metabolism
pgmB -4.1
Transporters & Efflux pumps
NMB0177 8.6
NMB2136 3.8
NMB0227 2.6
NMB1946 2.3
mtrD -11.7
cysP -7.7
NMB0548 -5.3
secD -4.0
NMB0696 -4.0
Membrane components
pilF 3.1
fHbp -8.5
htpX -7.7
yidC -6.5
comL -6.1
comE -4.2
GNA2091/YraP -1.7
Iron Storage & Transport
bfrB 3.9
bfrA 3.1
fbpA 1.5
Histidine metabolism
hisD 1.8
hisB -1.9
Ribosomal proteins & biogenesis
rplU 22.9
rplS 3.7
rplV 3.3
rpmB 2.9
rplD 2.3
NMB0475 2.4
rpmD -36.0
rimP -6.5
rplT -1.4
rpsL -1.2
rpsM -1.2
Queuosine biosynthesis
queF 4.5
Oxidoreductases
NMB1796 2.6
prx 1.7
tehB -2.6
Amino acid metabolism
NMB0337 3.3
NMB1428 3.0
prlC 1.7
leuC -7.5
pheA -4.9
dapE -3.0
aroG -2.5
dapD -1.5
DNA replication/repair & restriction/ modification
hsdM 3.0
NMB1580 2.3
NMB1368 -5.7
topA -5.1
ssb -5.0
gyrA -1.5
Co-factor & vitamins metabolism
thiD 2.0
ribH -10.1
suhB -4.0
panC -2.5
rpmD -2.5
ribA -1.3
Chaperon proteins
Lon 2.4
ppiB 2.4
dnaJ -2.0
surA -2.2
groS -2.2
fkpA -1.6
Unknown
NMB1584 /mmsB? 38.9
NMB1599 11.9
NMB0650 9.4
NMB0865 9.3
NMB0866 6.8
NMB0649 4.0
NMB1600 3.7
NMB1406 3.5
NMB0859 2.9
NMB0861 2.5
NMB1059 1.7
NMB1084 1.7
NMB0086 -5.2
NMB0039 -3.0
